# Advances in the relationship between ferroptosis and epithelial–mesenchymal transition in cancer

**DOI:** 10.3389/fonc.2023.1257985

**Published:** 2023-11-07

**Authors:** Wenrong Mu, Zubang Zhou, Liping Shao, Qi Wang, Wanxue Feng, Yuling Tang, Yizong He, Yuanlin Wang

**Affiliations:** ^1^ The First Clinical Medical College of Gansu University of Chinese Medicine, Gansu, China; ^2^ Department of Ultrasound, Gansu Provincial Hospital, Gansu, China

**Keywords:** ferroptosis, epithelial-mesenchymal transition, chemoresistance, mechanism, malignancy

## Abstract

Epithelial-mesenchymal transition (EMT) is a cellular reprogramming process that converts epithelial cells into mesenchymal-like cells with migratory and invasive capabilities. The initiation and regulation of EMT is closely linked to a range of transcription factors, cell adhesion molecules and signaling pathways, which play a key role in cancer metastasis and drug resistance. The regulation of ferroptosis is intricately linked to various cell death pathways, intracellular iron homeostasis, and the protein network governing iron supply and storage. The ability of ferroptosis to disrupt cancer cells and overcome drug resistance lies in its control of intracellular iron ion levels. EMT process can promote the accumulation of iron ions, providing conditions for ferroptosis. Conversely, ferroptosis may impact the regulatory network of EMT by modulating transcription factors, signaling pathways, and cell adhesion molecules. Thus, ferroptosis related genes and signaling pathways and oxidative homeostasis play important roles in the regulation of EMT. In this paper, we review the role of ferroptosis related genes and their signaling pathways in regulating cancer EMT to better understand the crosstalk mechanism between ferroptosis and EMT, aiming to provide better therapeutic strategies for eradicating cancer cells and overcoming drug resistance.

## 1 Introduction

Recently, the incidence of cancer has increased dramatically, making it the second most common cause of death in the world. EMT (EMT) is a major cause of cancer metastasis and characterized by proliferation, division, invasion and migration. Therefore, there is a need for an effective EMT treatment strategy, which can be broadly classified into surgical and nonsurgical approaches (1). Most conventional nonsurgical treatments are closely related to apoptosis, particularly those associated with the cystine aspartate protease (caspase) family (2). However, the cancer cells exhibit apoptosis resistance due to the negative effects of over-expression of protein and the escape of apoptosis due to the mutation of apoptosis and multiple drug resistance, and resulting in loss of therapeutic efficacy of conventional chemotherapy (3). Thus, there is an urgent need to develop new anticancer tools to overcome these limitations and improve cancer outcomes.

In 2012, a novel programmed cell death form distinct from necrosis and apoptosis, called ferroptosis, was discovered (4). Ferroptosis is a complicated biological process, which involves the disruption of iron metabolism and the accumulation of ROS (ROS). It can be controlled through co-regulatory mechanisms and specific signaling pathways, where cell-to-cell contact plays a crucial role in ferroptosis regulation (5). Recent research indicates that ROS, DNA damage and metabolic reprogramming are needed to activate lipid peroxidation and ferroptosis (6). The main reasons for ferroptosis are the abnormal increase of iron-dependent lipid free radicals and the imbalance of oxidation-reduction homeostasis (7). The oxidation-reduction basis of ferroptosis is primarily achieved through the inactivation of cellular antioxidant defense systems, Among them are cystine/glutamate reverse transport system Xc−, glutathione (GSH), glutathione peroxidase 4 (GPX4), and FSP1 (8). system Xc−, which is a protein carrier for small molecule in ferroptosis, is made up of the regulatory subunit solute carrier family 3 member 2 (SLC3A2) and the catalytic subunit solute carrier family 7 member 11 (SLC7A11). It helps extracellular cystine to enter the cell membrane and synthesize GSH via ATP-dependent enzymes such as glutamate cysteine ligase and GSH synthetase. It also exports intracellular glutamate to the extracellular fluid through the system Xc− to maintain and protect normal cell function (9). SLC7A11 is regulated by a variety of factors, such as TP53, NFE2L2, and BRCA1-associated protein 1, Mucin 1, as well as cell-surface associated proteins or autophagy-associated genes, for example, BECN1. Inhibition of SLC7A11 pathway may be an important upstream mechanism for ferroptosis. Thus, the survival and growth of cancer cells are largely dependent on the transport activity of the system Xc−, which makes them a potential target for the development of anticancer drugs. GSH, a powerful antioxidant, is critical in inhibiting the formation of peroxides and the reduction of ROS and reactive nitrogen in cell membranes. As a cofactor and substrate of GPX4, it can prevent the lipid peroxidation in the biological membrane. Lipid peroxidases are responsible for the death of cells and serious oxidative damage (10). In addition, FSP1, a newly developed antioxidant, activates coenzyme Q10 to remove lipid peroxidation. Therefore, lipid peroxidation and ferroptosis can be inhibited at the same time (11). During ferroptosis, membrane damage occurs. But cells can reduce the degree of damage to the membrane by activating GSH, coenzyme Q10, Nuclear factor erythroid 2 (Nrf2) and membrane repair pathway (ESCRT-III pathway) (12). Furthermore, GPX4, a phospholipid peroxidase, plays an important role in reducing phospholipid peroxides and inhibiting the reaction between phospholipid peroxides and labile iron pools in cells through depletion of GSH. This, in turn, terminates the free radical-driven amplification reaction. Therefore, GPX4 not only acts as anti-lipid peroxidation, but also acts as a downstream regulator that inhibits ferroptosis (13). Erastin and RSL3 are two typical inducers of ferroptosis. Erastin triggers ferroptosis by inhibiting the system Xc−, leading to cysteine depletion and insufficient synthesis of GSH, disrupting the function of GPX4 (14). Recent studies have also shown that certain drug-resistant cancer cells with characteristics of EMT are highly sensitive to ferroptosis inducing drugs, providing a new research area for targeted therapy (15).

In the course of EMT, epithelial cells lose their apical-basal polarity and cell-cell adhesion, and acquire spindle-shaped mesenchymal shapes to gain the ability to migrate and invade, which are closely related to the invasion, migration and metastasis of cancer (16). During the course of EMT, the cells lost their epithelial cell markers, such as E-cadherin (CDH1), cytokeratins, and occludin, and upregulates N-cadherin, vimentin, fibronectin, β1 and β3 integrins (17). EMT is related to core transcription factors, epigenetic regulation, post-transcriptional regulation, and gene mutation. ZEB1/2, SNAIL1/2 and TWIST1/2 are key transcription factors (18). Epigenetic modification plays an important role in EMT through regulation of transcription factors. There is a positive correlation between high methylation of CDH1 and EMT (19). CDH1 is a subtype of epithelial cell specific calcium binding proteins (E-cadherin), which is expressed in epithelial cells. As a tumor suppressor gene, E-cadherin plays a role in regulating cell polarity, differentiation, migration, and stem cell properties (20). Furthermore, ZEB1/2 binds to the E-box regulatory gene sequence in the promoter to repress CDH1 transcription and activate the expression of mesenchymal phenotypes such as wave proteins, thereby increasing cell proliferation and metastasis (20). ZEB1 also regulates the expression of CDH1 via epigenetic regulation, which is critical to EMT via MAPK/ERK, JAK/STAT3, PI3K/AKT, and so on (18). The expression of SNAIL is up-regulated in recurrent tumors by binding to E box on CDH1 promoter and inhibiting CDH1 transcription (20). TWIST can decrease the expression of E-cadherin, and enhance the expression of Vimentin, N-cadherin, which may be related to the prognosis of cancer (21). Some non-coding RNAs, such as miR-200 and miR-205, regulate the expression of EMT-induced transcription factors, which can inhibit ZEB1/2 expression. Inhibition of miR-200c or high expression of ZEB1 may lead to poor prognosis in various cancers, including breast and ovarian carcinoma. In the regulation of EMT, long non-coding RNAs (lncRNAs), for example, TGF-β-activated lncRNA (lncRNA-ATB) and lncRNA-PNUTS. They can act as sponges for miR-200 and miR-205, preventing them from inhibiting the expression of EMT-inducing transcription factors (22). TGF-β, a well-defined epithelial cell inhibiting growth factor, can induce EMT, resulting in the loss of E-cadherin and the proliferation of mesenchymal markers, such as N-cadherin (23). Mutations of TGF-β can cause cancer cells to proliferate uncontrollably. Successfully promoting the migration and metastasis of cancer cells by regulating the expression of EMT transcription factors. Therefore, these transcription factors can serve as therapeutic targets in cancer treatment. It is hoped that further study of these mechanisms will lead to a new breakthrough in the treatment of cancer and improve the prognosis of cancer patients.

Ferroptosis is closely related to EMT, which are important biological processes associated with the growth of cancer. The latest research indicates that drug-resistant cancer cells that have Patients undergoing EMT are more susceptible to ferroptosis, indicating that they are more prone to being killed by ferroptosis inducers compared to non-drug-resistant cancer cells (15). It also suggests that ferroptosis may inhibit EMT progression in cancer.

Understanding the interaction of EMT with ferroptosis is critical to overcoming the challenges of cancer therapy (3). It is imperative to find new treatments to fill the gaps in current therapeutic strategies and to improve the overall outcome of patients. Future studies should explore the molecular mechanisms and interactions of EMT and ferroptosis in order to advance the field of cancer therapy.

## 2 EMT regulation in cancer by ferroptosis-associated genes and their signaling pathways

### 2.1 GPX4 Inhibition

Recently, it has been demonstrated that high mesenchymal status is closely related to GPX signaling. GPX4 is involved in the induction of ferroptosis in cancer cells. The EMT-mediated transcription factor ZEB1 regulates and induces EMT via TGF-β/Smad, Furthermore, it can promote ferroptosis through direct inhibition of the activity of GPX4 and activation of the PPARγ (PPARγ) pathway (24). TGF-β can promote EMT of cancer cells by inducing ZEB1 and enhancing GPX4 inhibitor activity (25). Metadherin (MTDH), a newly discovered cancer-related antigen, promotes EMT, invasion and metastasis in a wide range of cancers, including breast and colon. It downregulates E-cadherin expression through activation of Wnt/β-catenin, MAPK and PI3K/AKT (26). MTDH, as an RNA binding protein and is also a positive regulator of EMT, can make cells more sensitive to ferroptosis by decreasing GPX4, SLC3A2, and reducing cysteine, GSH and increasing glutamate (24). The regulation of MDTH-GPX4/SLC3A2-GSH axis may contribute to the improvement of EMT susceptibility to ferroptosis. Moreover, it has been shown that targeting GPX4 can inhibit the growth and metastasis of epithelial-derived carcinoma by inhibiting the proliferation of steroid regulatory element-binding protein (27). Furthermore, Jiang et al. (28) have shown that targeting GPX4 might be a useful strategy to overcome the first generation NSCLL. Loss of GPX4 may result in the death of iron in the refractory carcinoma cells due to various cancers and therapies. Thus, targeting GPX4 or inducing ferroptosis might be an effective therapeutic strategy to overcome drug resistance.

It has been shown that targeting GPX4 or inducing ferroptosis may be an effective way to overcome drug resistance. Targeted regulation of GPX4 is promising as a novel approach to cancer therapy. The further study of the role of GPX4 in the development and therapy of cancer will contribute to a better understanding and application of this strategy.

The CD44-dependent increase in iron endocytosis promotes iron-dependent demethylase activity, which promotes the expression of genes associated with EMT signaling, thereby sensitising cancer cells to iron transfer (29). Data from these preclinical studies suggest that EMT may confer sensitivity to ferroptosis-based treatments.

### 2.2 Nrf2/NFE2L2

Nrf2 is a transcription factor with a basic leucine zipper domain that partially supports tumor cell proliferation by activating the oxidative stress response (30). rf2 regulates ferroptosis directly or indirectly by regulating the content of GPX4, Intracellular free iron level, the regeneration of coenzyme NAD (P) H and mitochondrial function (14). NF2 has an adverse effect on ferroptosis by increasing the expression of Fe and ROS, such as quinoline oxidoreductase 1 and hemolymph 1(HO-1) (31). The Kelch-like ECH-associated protein 1 (Keap1) regulates the activity of Nrf2 in the cytoplasm, which is critical for ubiquitination and proteasome degradation (30). Using a protein–protein interaction (PPI) network, It has been shown that Nrf2 regulates ferroptosis by directly influencing the GPX4 and PPARγ pathways, and regulates the level of iron ions via Nrf2/HO-1 (14). High stromal states make many cancer cells less sensitive to ferroptosis, while drug-resistant stromal states help cells to avoid ferroptosis by regulating lipid peroxidation (24). Moreover, GPX4 inhibition leads to intracellular iron-mediated peroxidation, inducing ferroptosis. Therefore, ferroptosis induction can effectively eliminate EMT in cancer (32). The Keap1/Nrf2/HO-1 signaling pathway has been found to play an important role in the regulation of ferroptosis and EMT (33).

Nrf2 binds directly to the anti-oxidation reaction element region of the system Xc− Subunit promoter, and promotes the expression of the system Xc− (34). Inhibiting Nrf2 or over-expression of Keap1 reduces the level of system Xc−, whereas over-expression of system Xc− has no effect on Nrf2 and Keap1. Thus, the system Xc− may be a potential downstream target of Nrf2 (35). Atorvastatin inhibits the system Xc−/GPX4/Nrf2 axis by interfering with the antioxidant system and promoting ferroptosis. Inhibition of system Xc−, GSH depletion, and increased oxidative stress all result in the occurrence of EMT. Therefore, the control of the system Xc− can inhibit the EMT, thereby reducing the metastasis and resistance of the cancer (36).

Nrf2 plays a key role in the regulation of EMT. Studying Nrf2, as well as its downstream targets, will help to better understand the mechanisms involved and guide the development of new therapeutic strategies. Deep study of Nrf2’s function and regulation network will be helpful to enhance the efficacy of cancer therapy, particularly in overcoming therapeutic resistance and inhibiting metastasis.

### 2.3 BTB and CNC homologue 1 (BACH1)

BACH1 is a hemoglobin-binding transcription factor, which plays an important role in the regulation of oxidative stress, hemoglobin, and iron metabolism (37). BACH1 regulates EMT through modulation of intercellular adhesion genes, such as claudin3 and claudin4. It also regulates the transcription factors, including FOXA1 and Snail2, which may be involved in the EMT, and form a direct effect on the EMT. BACH1 inhibits the expression of CDH1 and promotes EMT, whereas FOXA1 acts as an intermediary between BACH1 and E-cadherin (38). In addition, BACH1 directly activates Snail2, which is a typical transcription factor that activates EMT through inhibition of adhesion and promotion of stem cells (38). Moreover, BACH1 can promote ferroptosis through inhibition of GSH pathway and instability of iron metabolism (37). Through the activation of Hippo signaling pathway, E-cadherin-mediated cell contact can inhibit ferroptosis, thus decreasing the activity of YAP, the transcription coactivator, caused by ferroptosis (39). YAP is activated during EMT initiation, resulting in increased susceptibility to ferroptosis. Since BACH1 can inhibit E-cadherin expression and cell adhesion, it can be used to inhibit E-cadherin-Hippo-YAP pathway via CYC-GSH-GPX4 to promote ferroptosis (37).

BACH1 can also inhibit Nrf2 signaling pathway by binding to Nrf2. Deletion of BACH1 increased expression of Nrf2-regulated genes, especially HO-1 (40). BACH1 promotes the increase of unstable iron content in the cell, whereas Nrf2 inhibits an increase in intracellular iron content. Increasing the ratio of BACH1/Nrf2 leads to ferroptosis (41). Therefore, BACH1 can be used to link ferroptosis to EMT, leading to a deeper linkage.

BACH1 plays an important role in the regulation of EMT. Further research on the function and regulation of BACH1 will help us better understand the interplay between EMT and ferroptosis and provide guidance for the development of new therapeutic strategies. At the same time, the BACH1/Nrf2 regulatory networks are being studied, and their potential applications in cancer therapy are discussed.

## 3 Role of ROS in the relationship between ferroptosis and EMT

ROS are highly reactive molecules that are important regulators of signaling pathways. They are tumorigenic in nature and act as secondary messengers in the cellular signaling cascade to induce and maintain the oncogenic phenotype of cells as well as sustain cancer development by inducing DNA damage and promoting the proliferation, survival, and migration of tumor cells (42). ROS acts as a signaling molecule capable of triggering different types of cell death, including ferroptosis, which requires ROS accumulation throughout the process, thereby disrupting cell membrane integrity (4). A study has reported that ROS generation is enhanced during EMT (43). In general, ROS are produced via a series of specialized enzyme complexes or as a by-product of redox reactions. Furthermore, in the Fenton reaction, ROS are produced in lysosomes and unstable iron pools where ferritin phagocytosis occurs. Ferroptosis induction involves ROS production, and ferritin phagocytosis-mediated ROS production contributes to EMT inhibition. Therefore, the relationship between EMT and ferroptosis and ROS production should be elucidated. Dithiocarbamate derivatives are a class of iron-chelating agents with anticancer properties, including the ability to induce ferritinophagy (44). Deng et al. (33) reported that 2,2’-dipyridylketone hydrazone dithiocarbamate (DpdtbA) is an antagonist of TGF-β1-induced EMT by the generation of ROS induced by ferritinophagately. In addition, DpdtbA treatment led to the removal of GPX4 and the system Xc−, which increased lipid peroxidation, indicating that DpdtbA mimics the role of the ferroptosis inducer Erastin in the induction of ferroptosis (44). Experimental results also showed that Fer-1 was able to counteract the regulation of DpdtbA on Vimentin and E-cadherin, suggesting that the ferroptosis induction was involved in suppressing EMT (33). Nevertheless, cancer cells thrive in hypoxic environments, and DpdtbA induces EMT suppression under these conditions, possibly associated with activation of P53, PHD2/Hif1α pathways (45).

ROS plays an important role in the development of cancer and may affect EMT and ferroptosis. As iron chelators, dithiocarbamate derivatives, such as DpdtbA, can interfere with the generation of ROS induced by ferritinophagy and may inhibit EMT. Understanding this mechanism will be helpful in the development of cancer metastasis and drug resistance, and in the development of therapeutic strategies. The exploration of ROS, EMT, and ferroptosis signaling pathways will provide a better understanding and strategy for cancer therapy.

### 3.1 ROS regulatory factor - p53

P53 is a tumor suppressor protein and a key and complex ROS regulatory factor in cancer development. It has both antioxidant and pro-oxidant functions (45). It has both antioxidant and pro-oxidant functions. P53-inducible ROS may induce cell death via apoptosis and ferroptosis, and its anti-oxidative activity may contribute to the inhibition of the growth of cancer by preventing DNA damage (46). Activated P53 binds to SLC7A11’s promoter region, inhibits its transcription activity, decreases the intracellular cysteine and GSH concentrations, and enhances the accumulation of ROS, Indirect inhibition of the activity of GPX4, resulting in the formation of ferroptosis in cells (47). Studies of cambogia (an iron chelator) have demonstrated that it can markedly increase the levels of lipid ROS in TGF-β1 induced melanoma cells, Regulation of oxidative stress by activating P53/SLC7A11/GPX4 signaling pathway, induction of ferroptosis and inhibition of EMT, And thus block the invasion and metastasis of the tumor (48). The PI3K/AKT/mTOR pathway is critical for the growth and survival of cells, and the inhibition of this pathway may increase the therapeutic effect of ferroptosis. There is growing evidence that the AKT/mTOR pathway is involved in the regulation of critical processes like EMT, motility, and metastasis of cancer cells (49, 50). The AKT/mTOR pathway is downstream of P53, sActivation of P53 results in AKT and mTOR downregulation. It was found that the EMT reversal induced by 2,2 ‘-dipyridyketone hydrazine dithiocarbamate was related to P53/AKT/mTOR pathway (23). Thus, through modulation of P53/PI3K/AKT/mTOR signaling pathway, it is possible to regulate the susceptibility of cancer cells to ferroptosis.

P53 plays an important role as a regulator of ROS in the development of cancer. It influences the proliferation, survival and EMT of cancer cells through induction of ROS and regulation of PI3K/AKT/mTOR. Deep Research on the mechanism of P53 in regulating ROS, ferroptosis, and EMT will help to better understand the molecular mechanisms underlying cancer progression and provide new insights into the development of therapeutic strategies that target these pathways.

### 3.2 ROS regulatory factor – Nrf2

Nrf2 is an important factor in the regulation of ROS. When ROS levels increase, the degradation of Nrf2 is disrupted by Keap1, allowing Nrf2 to stabilize and enter the cell nucleus to activate a protective antioxidant response (51). By inducing iron engulfment within ferritin, the activation of Nrf2 results in the up-regulation of HO-1, which is advantageous to induce ferroptosis (52). These results suggest that Keap1/Nrf2/HO-1 can regulate the EMT and ferroptosis processes. Generally speaking, the intensity of ROS induced by ferritin engulfment has a great effect on the occurrence of ferroptosis and progression of EMT, and also influences the activation of Keap1/Nrf2/HO-1 (53).

Deep research on the role of Nrf2 in the regulation of ROS, ferroptosis, and EMT may help to understand the complexity of cancer development and therapy. As one of the regulatory mechanisms produced by ROS, iron sequestration plays an important role not only in the induction of ferroptosis, but also in the regulation of EMT and activation of Keap1/Nrf2/HO-1 pathway through the influence of ROS. Therefore, further research on the mechanism of Keap1/Nrf2/HO-1 signaling pathway will contribute to the development of new therapeutic strategies, especially in the treatment of cancer.

### 3.3 ROS-regulated pathway - Wnt/β-catenin signaling pathway

The Wnt/β-catenin signaling pathway plays an important role in the development and progression of cancer by regulating cell proliferation and apoptosis. When Wnt ligands (such as Wnt3a and Wnt1) are secreted, they bind to Frizzled receptors and low-density lipoprotein receptors, thereby blocking the pathway of β-catenin degradation and activating downstream gene transcription Cyclin D1, and WISP (54). These genes are activated to promote the EMT, thus triggering the growth and metastasis of the tumor (55). In the activation process of the Wnt signaling pathway, β-catenin enters the cell nucleus and binds to transcription factors TCF/LEF. TCF4 plays an important role in Wnt/β-catenin signaling pathway (56). It is suggested that the activation of Wnt/β-catenin signaling pathway enhances TCF4 expression and promotes GPX4 transcription via β-catenin/TCF4. Thus, the formation of ROS, and the inhibition of ferroptosis in gastric carcinoma cells were inhibited (57). The Wnt signaling pathway plays an important role in the regulation of the progression of EMT and ferroptosis. At present, however, there is no evidence to support the specific association of Wnt signaling with ferroptosis and EMT. Therefore, further study on the role of ferroptosis in the regulation of EMT via Wnt signaling pathway will contribute to a better understanding of the interaction between the two pathways in cancer growth and metastasis.

### 3.4 The role of autophagy in ROS

Autophagy is a cellular self-digestive process that maintains cellular homeostasis and function by degrading and recycling harmful or ageing substances within the cell. Recent studies have found that autophagy is closely related not only to cell survival and death, but also to some important biological processes such as ferroptosis and EMT (58).

**Figure 1:**
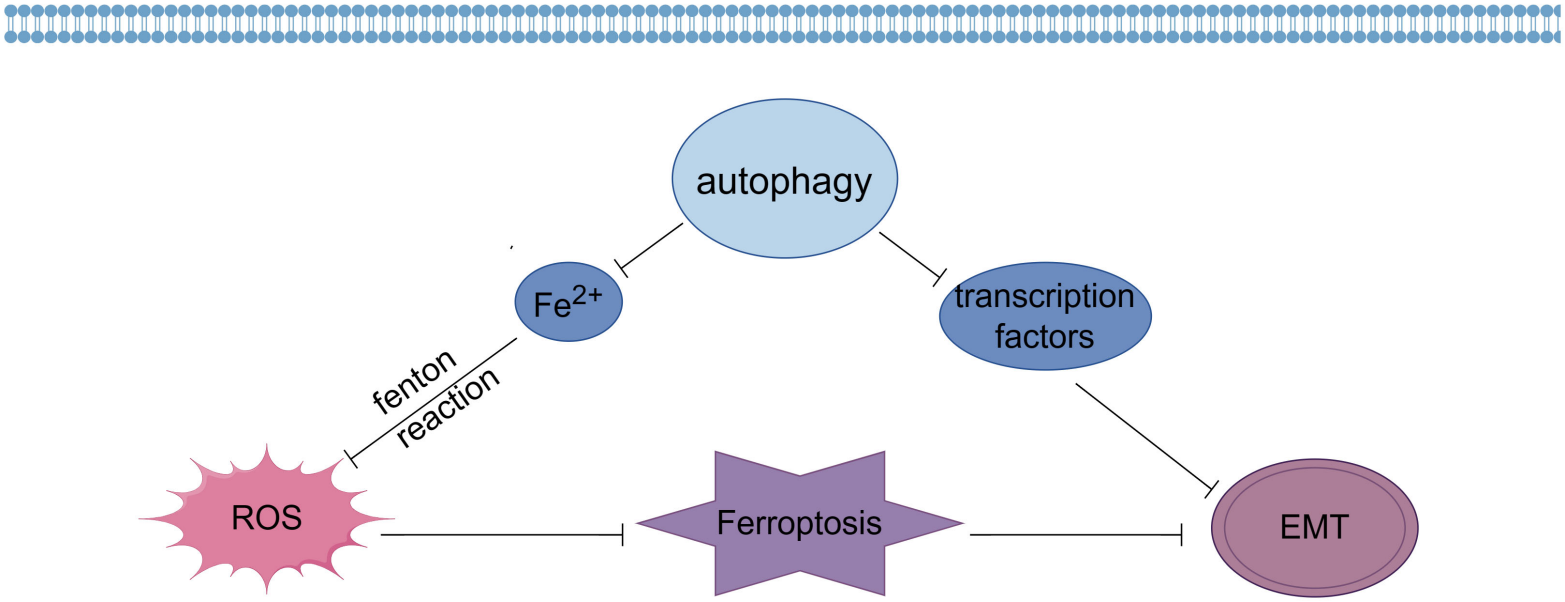
Effects of autophagy on ferroptosis and EMT (By Figdraw).

ROS-induced autophagy is a process that gives expression to key regulators of ferritin degradation and transferrin receptor 1 during ferroptosis, which has been suggested by recent studies to be a process of autophagic cell death that can be termed autophagy-dependent ferroptosis (59, 60). Firstly, autophagy affects the occurrence of ferroptosis by regulating ROS generation (**Figure 1**). Studies have shown that autophagy can remove excessive intracellular iron ions and reduce the reaction between iron ions and ROS, thereby reducing the amount of ROS generation and thus alleviating the oxidative stress state of cells (61). In addition, autophagy can also reduce intracellular ROS generation by removing sources of ROS generation, such as mitochondria and endoplasmic reticulum. Thus, autophagy can prevent ferroptosis by regulating ROS generation (62).

Secondly, autophagy can also influence the onset of ferroptosis by regulating the clearance of ROS. It was found that autophagy can remove intracellular ROS and reduce oxidative stress in cells (63). Autophagy reduces intracellular ROS content by degrading ROS-generating sources such as mitochondria and transporting ROS to lysosomes for degradation through the autophagosome membrane. In addition, autophagy can enhance ROS scavenging by regulating the expression and activity of antioxidant enzymes. Thus, autophagy may also inhibit ferroptosis by regulating ROS scavenging (64).

In addition, autophagy is also closely related to the occurrence of EMT (**Figure 1**). Recent studies have shown that autophagy can inhibit EMT by degrading and regulating the expression of EMT transcription factors such as Snail, Twist and ZEB (65). In addition, autophagy can also influence the occurrence of EMT by removing intracellular aging substances and harmful substances and maintaining cellular homeostasis and function (66).

Taken together, ROS is involved in various response pathways affecting tumorigenesis, and a comprehensive understanding of the regulation of different cellular responses can facilitate the clinical application of ROS or ROS-regulated pathways to formulate and improve the shortcomings of current cancer therapies.

**Figure 2:**
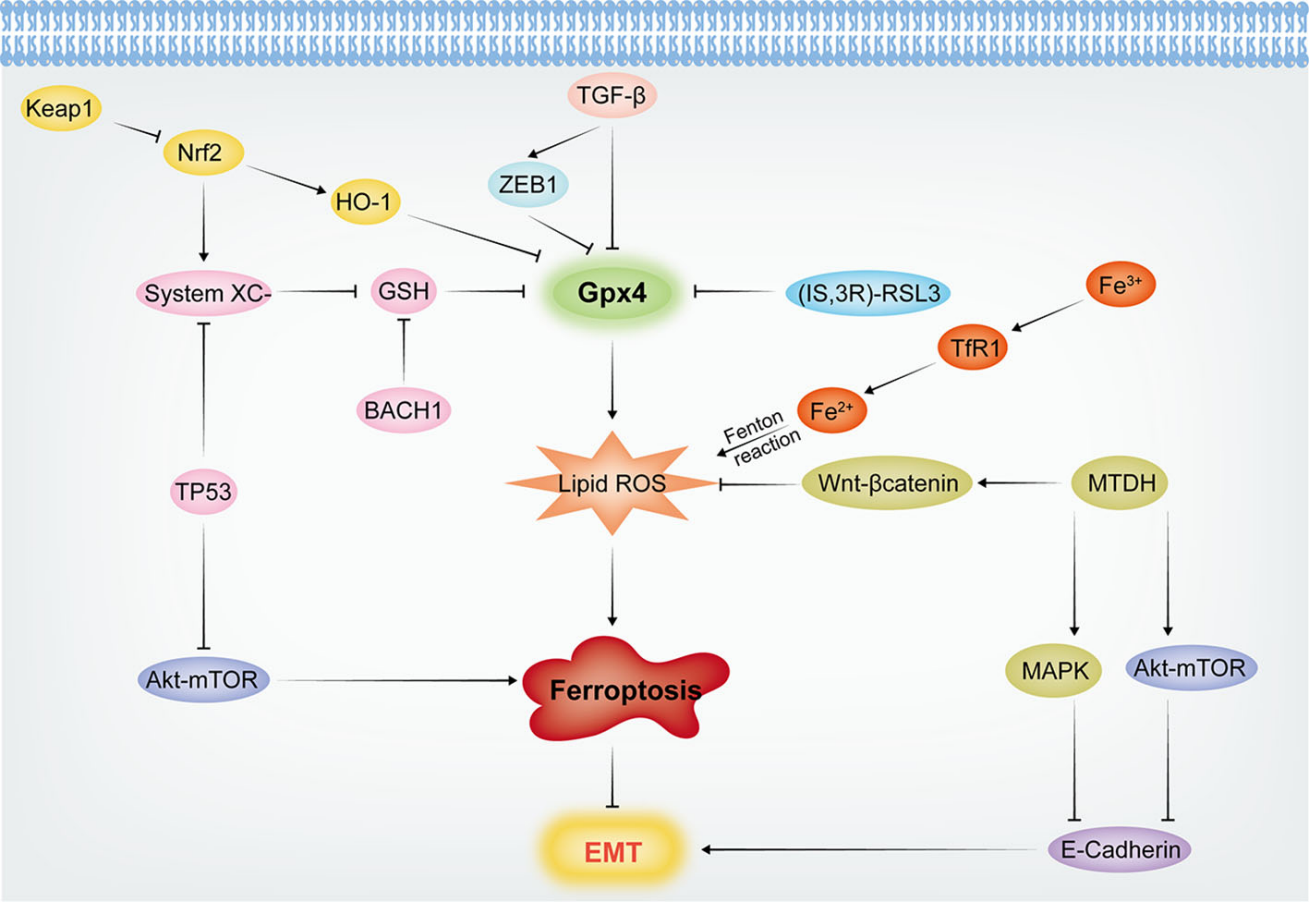
The mechanisms of Ferroptosis and EMT crosstalk.

Therefore, the above ferroptosis related genes and their signaling pathways can be regulated to modulate EMT occurrence in cancer cells (**Figure 2**), providing more strategies for tumor treatment.

## 4 Conclusion

Significant progress has been made in the field of tumor treatment in recent years, including the increasing use of targeted drugs and immunotherapy. However, the problem of tumor drug resistance remains challenging. Therefore, reversing or inhibiting EMT has become a potential cancer treatment strategy to suppress the migration and metastasis of tumor cells to distant sites. Resistant cancer cells, upon transition to a mesenchymal state, become resistant to apoptosis induced by conventional therapies but are highly sensitive to ferroptosis inducers due to changes in their metabolic state. By inhibiting or activating ferroptosis related genes and their signaling pathways, the regulation of EMT by ferroptosis can be achieved, thereby improving the prognosis and treatment outcomes of cancer and providing new strategies to address clinically challenging cancers.

Further research on the interaction mechanism between ferroptosis and EMT not only helps us gain a deeper understanding of the mechanisms of tumor metastasis and recurrence but also provides new strategies for tumor treatment. Exploring and confirming the deeper relationship between these two processes can help us better grasp the diversity and complexity of tumor development, thus guiding clinical practice and the development of treatment strategies. Ferroptosis and EMT have significant implications in cancer treatment. Reversing or inhibiting EMT and modulating genes and signaling pathways related to ferroptosis can bring about breakthroughs in tumor treatment. Further research will help uncover the interplay mechanisms between these two processes and provide a theoretical basis for the development of more effective cancer treatment strategies.

## Author contributions

WM: Writing – original draft. ZZ: Funding acquisition, Resources, Writing – review & editing. LS: Writing – review & editing. QW: Writing – review & editing. WF: Writing – review & editing. YT: Writing – review & editing. YH: Writing – review & editing. YW: Writing – review & editing.
